# Visual consequent stimulus complexity affects performance in audiovisual associative learning

**DOI:** 10.1038/s41598-022-22880-z

**Published:** 2022-10-22

**Authors:** Kálmán Tót, Gabriella Eördegh, Ádám Kiss, András Kelemen, Gábor Braunitzer, Szabolcs Kéri, Balázs Bodosi, Attila Nagy

**Affiliations:** 1 Department of Physiology, Faculty of Medicine, University of Szeged, Dóm Tér 10, Szeged, 6720 Hungary; 2 Faculty of Health Sciences and Social Studies, University of Szeged, Szeged, Hungary; 3 Department of Applied Informatics, University of Szeged, Szeged, Hungary; 4 Nyírő Gyula National Institute of Psychiatry and Addictions, Budapest, Hungary

**Keywords:** Cognitive neuroscience, Learning and memory

## Abstract

In associative learning (AL), cues and/or outcome events are coupled together. AL is typically tested in visual learning paradigms. Recently, our group developed various AL tests based on the Rutgers Acquired Equivalence Test (RAET), both visual and audiovisual, keeping the structure and logic of RAET but with different stimuli. In this study, 55 volunteers were tested in two of our audiovisual tests, SoundFace (SF) and SoundPolygon (SP). The antecedent stimuli in both tests are sounds, and the consequent stimuli are images. The consequents in SF are cartoon faces, while in SP, they are simple geometric shapes. The aim was to test how the complexity of the applied consequent stimuli influences performance regarding the various aspects of learning the tests assess (stimulus pair learning, retrieval, and generalization of the previously learned associations to new but predictable stimulus pairs). In SP, behavioral performance was significantly poorer than in SF, and the reaction times were significantly longer, for all phases of the test. The results suggest that audiovisual associative learning is significantly influenced by the complexity of the consequent stimuli.

## Introduction

Associative learning is a basic cognitive function, in which different stimuli, cues, and/or outcome events are coupled. This learning type includes cognitive tasks like probabilistic learning^1,2^, latent inhibition^3^ and sensory preconditioning^4^, and equivalence learning^5,6^. Equivalence learning is a specific kind of associative learning in which two discrete and often different percepts (antecedents) are linked together based on a shared outcome (consequent). A visually guided paradigm, the Rutgers Acquired Equivalence Test (RAET), was developed by Myers et al.^7^ to investigate equivalence learning in humans. The test is computer-based and divided into two main phases: the acquisition and the test phases. In the acquisition phase, the subject’s task is to associate two different visual stimuli based on feedback information about the correctness of the choices. This way, the rule of pairing is acquired. In the subsequent test phase, the subject must recall the already learned associations (retrieval) and build new, hitherto not seen but predictable associations (generalization or transfer). Regarding the neural correlates, both the original study of the Myers group^7^ and subsequent investigations^8–13^ demonstrated that the acquisition phase is linked to the fronto-striatal loops, while the test phase is linked primarily to the hippocampi and the medial temporal lobe^7,14–19^. The basal ganglia and the hippocampi are structures of key importance in equivalence learning, and they are also involved in multisensory processing^20–23^.

Several studies reported that stimulus complexity influences auditory guided associative learning and more complex stimuli cause better responses and greater cortical activation^24–26^. It has also been demonstrated in studies from the cellular to the behavioral level that responses are quicker and more precise in the case of multimodal stimuli^22,27–30^. A recent study by our research group^31^, applying a modified but structurally identical version of RAET, showed that the complexity of the applied visual stimuli could also strongly influence the efficiency of associative learning: simple visual stimuli (antecedents: white, light gray, dark gray and black circles; consequents: colorless triangle, square, rhombus, and concave deltoid) with restricted semantic and color information allowed significantly poorer equivalence acquisition than more complex stimuli (antecedents: cartoon faces of a woman, a man, a boy and a girl; consequents: green, yellow, red and blue fish) without such feature restrictions. However, stimulus complexity did not affect retrieval and generalization (transfer).

Given that the key neural structures associated with RAET also play a role in multisensory processing, the question arises whether the complexity of the applied visual stimuli can also influence the effectiveness of multisensory (audiovisually guided) associative learning. In this study, we sought to answer this question. For this, we used two audiovisual tests, both of which follow the RAET paradigm: SoundFace (SF) and SoundPolygon (SP). These tests have been developed in our laboratory. Both tests use sounds as antecedent stimuli, but SF uses cartoon faces and SP uses simple geometric shapes as consequents. That is, the consequent stimuli in SF (colored cartoon faces of a boy, a girl, a man and a woman) are relatively complex in the sense that they have well-defined, readily detectable and readily verbalizable distinctive features (e.g., colors, gender, age), while the consequents in SP lack such features. By comparing our volunteers’ performance on these tests, we sought to test if consequent stimulus complexity influences audiovisual associative learning at all. It is important to note that the goal of this study was not to analyze how the gradual extraction of the different features of the consequent stimuli influences audiovisual associative learning (and to learn this way which features are more important, and which are less important for audiovisual associative learning). Instead, we chose to use the cartoon faces of RAET and a set of completely different, simple, non-face stimuli that lack all the specific features associated with the cartoon faces merely to establish the lack or existence of an effect.

## Results

All 55 volunteers completed both tests. The analysis of their performance data is presented according to the two main phases of the test paradigm (acquisition and test).

### Acquisition phase

**Figure 1:**
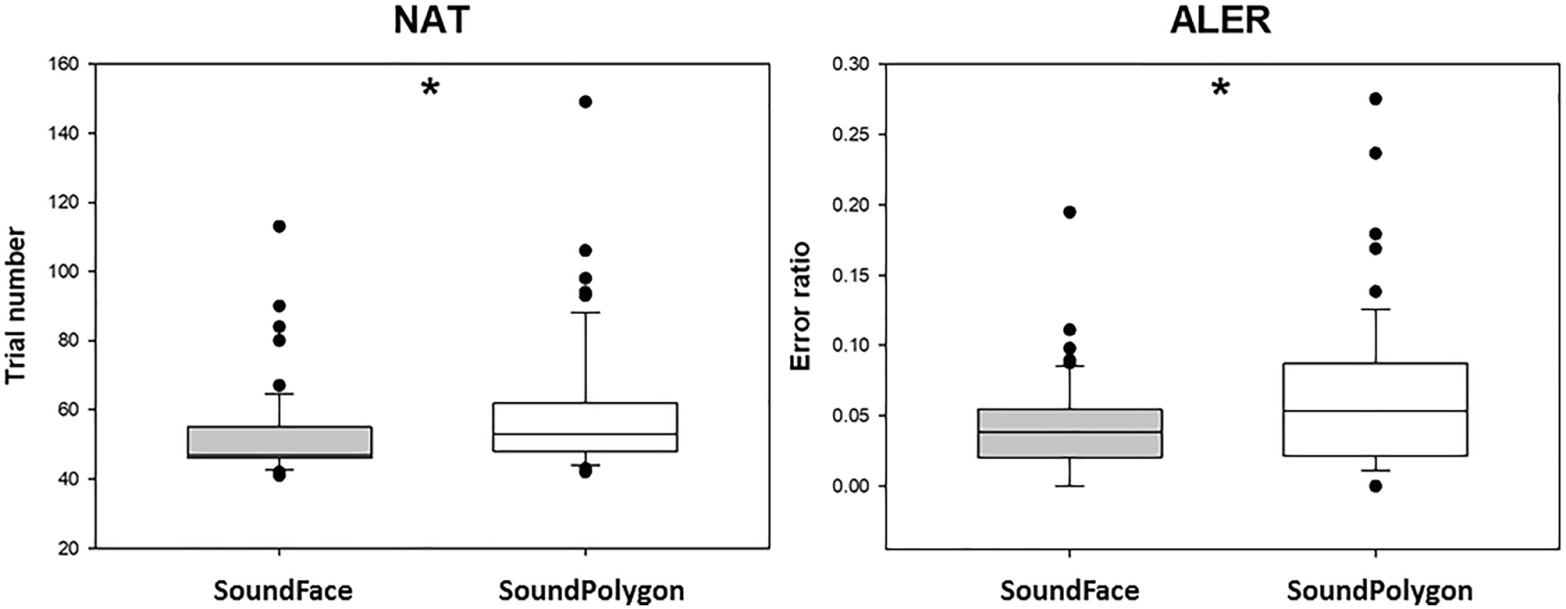
Performance in the acquisition phase in the two tests. NAT: the number of trials needed to complete the acquisition phase. ALER: error ratios in the acquisition phase. The lower margin of the boxes indicates the 25th percentile, the upper margin the 75th percentile, while the line within the boxes marks the median. The error bars (whiskers) above and below the boxes are the 90th and 10th percentiles, respectively. The dots over and under the whiskers represent the extreme outliers. Asterisk (*) indicates a significant difference at the level *p* < 0.05.

The median number of trials in the acquisition phase (NAT) in SF was 47 (range: 41–113), and in SP, it was 53 (range: 42–149). The participants needed significantly more trials to learn the associations in SP (*Z* = 2.417, *p* = 0.016) (Fig. 1).

In SF, the median of error ratios in the acquisition (ALER) was 0.038 (range: 0.00–0.19), and in SP, it was 0.058 (range: 0.00–0.28). The difference between the two tests was significant (*Z* = 2.213, *p* = 0.027) (Fig. 1). The median reaction time (RT) for the acquisition trials in SF was 1611.619 ms (range: 1095.022–4016.42), and in SP, it was 1834.810 ms (range: 1204.867–4762.33). The difference was significant (*Z* = 3.703, *p* = 0.0002).

### Test phase

**Figure 2:**
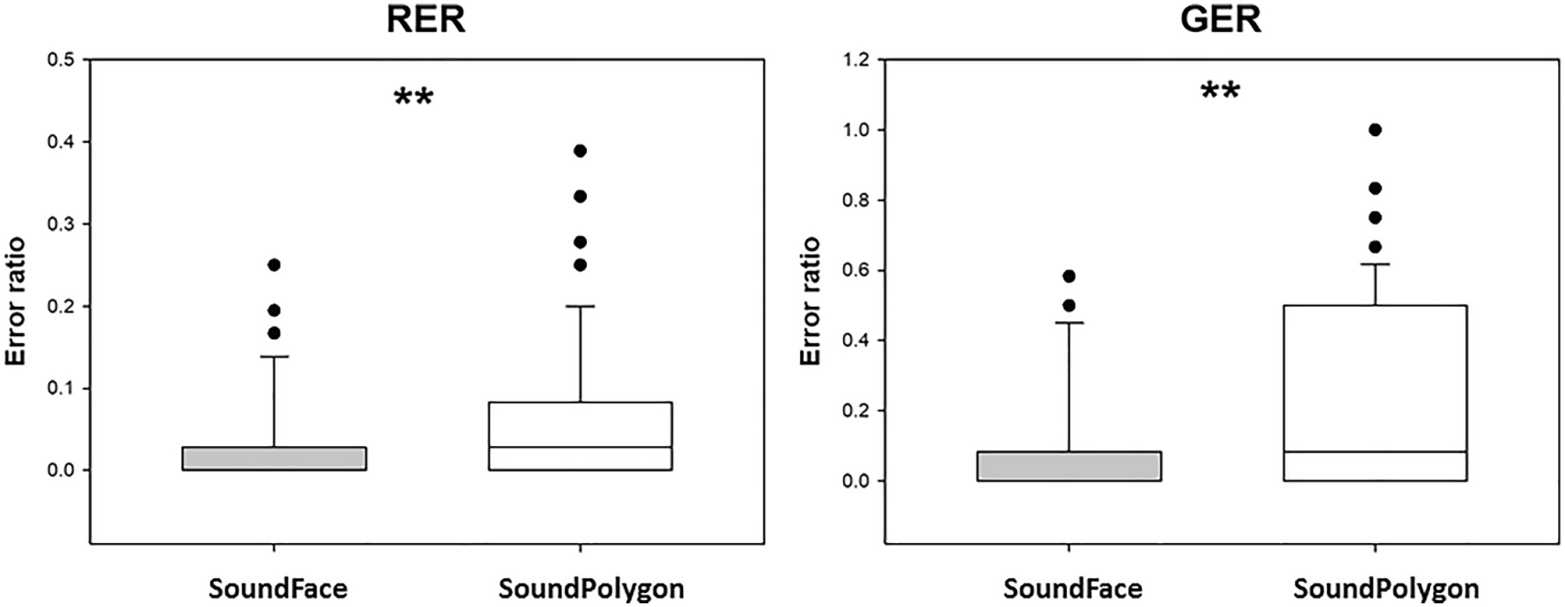
Performance in the test phase in the two tests. RER: retrieval error ratio, GER: generalization error ratio. Asterisk (*) indicates significant difference at the level *p* < 0.01. Otherwise, the conventions are the same as in Fig. 1.

The median of retrieval error ratio (RER) in SF was 0.00 (range: 0.00–0.25), and in SP, it was 0.028 (range: 0.00–0.39). The difference was significant (*Z* = 2.727, *p* = 0.0064) (Fig. 2). As for the reaction times, the median RT for the retrieval trials in SF was 1586.39 ms (range: 980.056–3802.11), and in SP, it was 2000.42 ms (range: 1222.33–4790.44). The difference was significant (*Z* = 4.994, *p* = 0.000001).

The median of generalization error ratio (GER) in SF was 0.00 (range: 0.00–0.58), and in SP, it was 0.083 (range: 0.00–1.00). The difference was significant (*Z* = 3.085, *p* = 0.002) (Fig. 2). The median RT for the generalization trials in SF was 1769.17 ms (range: 1145.75–5722.83), and in SP, it was 2544.25 ms (range: 1282.58–18,381.00). The difference was significant (*Z* = 3.938, *p* = 0.00008).

## Discussion

To our knowledge, this is the first study to investigate the effect of visual stimulus complexity on multisensory (audiovisual) guided associative learning. The same set of auditory stimuli were applied with two different series of visual stimuli in two tests based on the same paradigm. The two sets of visual stimuli differed in their feature richness and complexity. Multisensory guided equivalence learning and subsequent retrieval and generalization were all influenced markedly by the complexity of the visual stimuli. The difference also showed in significantly shorter reaction times when the more complex, feature-rich visual stimuli were used.

In this study, we used two audiovisual tests that were developed in our laboratory, based on RAET, a visually guided equivalence learning test designed by Catherine E. Myers and colleagues at Rutgers University^7^. The original paradigm tests visually guided pair learning, the retrieval of the already learned stimulus pairs and the ability to apply the previously learned associations to build new stimulus pairs. The key brain structures associated with this task (the hippocampi and the basal ganglia) are also known for their role in multisensory processing^22,23,32,33^. Therefore, we developed SF that uses cartoon faces as consequents with auditory antecedents^34^. SF was administered to healthy adult subjects and in psychiatric patient populations and the results were compared to those of the original, visually guided RAET test^13,34^. The comparison indicated that the fact alone that the task had become multisensory did not influence the volunteers’ performance to a significant degree, which led us to the conclusion that multimodality itself does not interfere with the efficiency of associative learning, retrieval, or generalization.

The visual stimuli both in the visual RAET and the audiovisual SoundFace are complex, colored stimuli with the potential to evoke associations and emotional responses, which, in turn, can serve as extra clues that recruit various cortical areas to enhance performance^35–37^. Such clues can thus mask the contribution of subcortical structures. We developed a new visually guided test, Polygon^31^ to reduce this effect. Polygon uses simple geometric shapes both as antecedents and consequents. Such simple shapes are relatively meaningless in themselves, and they can hardly evoke emotions. Therefore, we hypothesized that the use of geometric shapes would allow us to minimize cortical contributions to task performance and thus allow a better assessment of subcortical contributions. Indeed, the first study with Polygon^31^ revealed a specific pattern: it took significantly more trials for the volunteers to learn the stimulus pairs (and they made significantly more mistakes), but the reduced complexity of the stimuli had no significant effect on either the retrieval or the generalization part of the test phase.

The next logical step was to investigate what effect visual stimulus complexity might have on multisensory guided (audiovisual) equivalence learning. For this purpose, we combined the antecedent sounds of SF^31^ and the geometric shapes of Polygon^31^ into a new test (SP) and compared volunteers’ performance on this test to their performance on SF. This comparison is presented in this study. In contrast to what was found when tests using visual stimuli only were compared (RAET vs. Polygon)^31^, in the case of these multisensory tests, decreased stimulus complexity affected not only the acquisition phase, but the entire test phase, including retrieval and generalization. That is, it seems that when only visual stimuli are used, decreased stimulus complexity makes learning difficult, but if learning has been successful, retrieval and generalization are spared. Such a sparing does not seem to occur when visual stimulus complexity is decreased in an audiovisual (multimodal or multisensory) learning environment. While it comes as no surprise (in fact, it is somewhat intuitive) that stimulus complexity influences the efficiency of associative learning^24–26^, it is difficult to tell why decreased stimulus complexity affects performance in all phases of the audiovisual version of the test paradigm, while in the visual version, only acquisition is affected.

In an earlier study^38^, based on developmental data, we argued that learning and memory in this specific paradigm might be best described by the integrative encoding account of associative learning^39,40^. This account concentrates on two specific neural loops, the substantia nigra (SN)- striatum loop and the ventral tegmentum (VT)-hippocampus loop, which can be activated in parallel. While the SN- striatum loop supports primarily the voluntary learning of stimulus pairs with the help of feedback, the VT- hippocampus loop transfers information to the hippocampi, where a network of all encountered stimuli is constructed, with their connections and overlaps^41–46^. Then, this hippocampal network is activated in the test phase, which makes both retrieval and generalization possible. Based on this account, it is possible that the hippocampi, even if they are typically discussed as structures of key importance in explicit memory^39,47^, can support implicit functions as well. That is why, as we earlier argued^38,46^, children can generalize at a high level with poor acquisition and retrieval. In other words, they have the information, and they can use it as long as no conscious effort is involved. It must be noted that the integrative encoding account was developed based on visual (non-multisensory) learning paradigms, and we have no information whatsoever if it can be applied to multisensory learning as well. Based on the information available at this point, it might be hypothesized that decreased stimulus complexity affects all phases of the multisensory test (but not the visual test, as demonstrated earlier) because hippocampal compensation is either specific to the visual stimulus modality or it works only if stimuli of the same modality are used. This, however, is only a crude hypothesis, which is made by inference from the literature.

At the same time, it must be also noted that a direct comparison between SF/SP and the purely visual version of RAET is not possible as the latter uses colored cartoon fish as consequent stimuli. While it is not entirely obvious how this could contribute to the observed difference, the confounding effect of this methodological factor cannot be ruled out. Another possible explanation is that in SF, equivalence might be established between the face consequents too, based on their various features, which makes learning easier in SF than in SP, where consequents do not share such readily detectable features. However, assuming that the integrative encoding account^39,40^ (see above) can be applied in an audiovisual context too, this should affect only the acquisition phase. It may be that the lack of equivalence between the consequents in SP can explain poorer acquisition, but it does not seem to be a good explanation for poorer transfer. In the sense of the integrative encoding account, the fact that the stimulus pairs are more difficult to learn (which shows as more errors and a longer acquisition phase in RAET and its various versions) does not necessarily imply poor transfer. This is exactly what we saw when we administered the original version of RAET to small children.^46^.

Beside the poorer performance in all phases, reaction times were also significantly longer in SP. This is consistent with earlier findings, where, in a face-name association task, subjects’ reaction times were significantly shorter in response to more complex, high-salience colored faces expressing emotions than to less distinctive, grayscale face stimuli.^48^ This shows that complexity facilitates decision making, while in the (relative) lack of distinctive features, the facilitating effect is absent.

As for the limitations, we would like to point out the following.

First, this study is best understood as an exploratory study that sought to establish if the complexity of visual consequent stimuli has any effect on subjects’ performance in an audiovisual associative learning paradigm. By complexity, we simply meant how rich the applied stimuli were in well-described, readily identifiable (and possibly verbalizable) features that can be used as cues for learning. The cartoon faces are relatively rich in these: age, gender, hair color and facial expression are all such cues. In contrast, the polygons are colorless and they definitely do not have age, gender or any feature that is even close to a facial expression. This is a crude comparison, and it does not allow a finer analysis of the difference; all it allows is the conclusion that the presence or lack of such easily identifiable and obvious features does make a difference. Whether this is because these cues are easy to verbalize or because they are characteristically human features (which activate additional neural circuits) or for some other reason should be addressed in studies designed for that purpose. A logical next step would be to generate several sets of the cartoon face consequents with gradually decreasing complexity (cue content) and repeat the measurements with all the sets.

Second, it is a limitation of SP sepcifically that it is possible that the female voice and the female face are matched, which could provide an extra cue for learning. While it is not always the case (the stimulus pairings are randomly generated for each session), this could interfere with the results in some cases, even if not to a major extent.

In summary, in this study we have demonstrated that in a multisensory associative learning paradigm, where the antecedent stimuli are sounds, the complexity of the consequent visual stimuli has a significant effect on both learning and generalization.

## Methods

### Subjects

Fifty-five healthy adult volunteers participated in the presented study (27 females and 28 males, age mean: 31.36 ± 14.56 years, range: 18–69; five participants were over the age of 60). The estimated minimum sample size was 47, assuming *p* < 0.05, 1 − *β* = 0.95 and an effect size of 0.5. The sample size estimation was performed in G*Power 3.1.9.2 (Düsseldorf, Germany). The volunteers received no compensation and were free to quit without any negative consequence. The volunteers were informed about the study's background, goals, and procedures and gave their written informed consent. Any psychiatric, neurological, otological or ophthalmological condition that could interfere with the participant’s performance was an exclusion criterion. Before each testing session, the participants were shown the stimuli of the tests one by one (each stimulus once) to make sure that they could see and hear them correctly. The study protocol conformed to the Declaration of Helsinki in all respects and was approved by the Regional Research Ethics Committee for Medical Research at the University of Szeged, Hungary (27/2020-SZTE).

### The applied multisensory tests

Two audiovisual tests of our own development were administered (SoundFace and SoundPolygon, see below). Both tests were run on laptops (Lenovo ThinkBook 15-IIL, Lenovo, China), and the auditory stimuli were administered through over-ear headphones (Sennheiser HD439, Sennheiser, Germany). The volunteers were tested one-by-one in a quiet room, sitting at a comfortable distance (57 cm) from the screen. No forced quick responses were expected to avoid performance anxiety, but the participants were instructed to respond as quickly as possible. This way, explicit time pressure could be avoided, yet, the participants were aware that it was desirable that they spent a limited amount of time with each trial. The volunteers completed both tests immediately one after another, in a pseudorandom order to avoid the carry-over effect.

**Figure 3:**
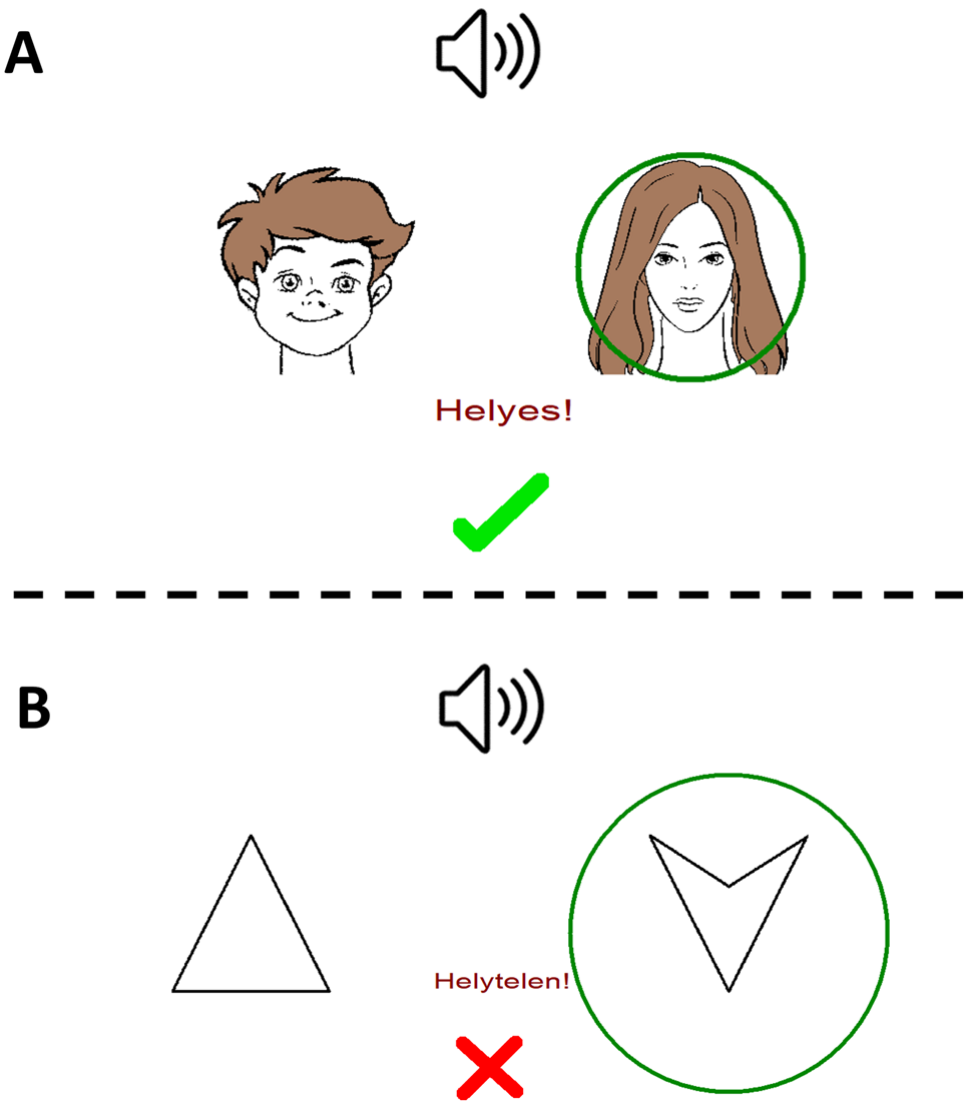
A trial in the acquisition phase of SoundFace (**A**) and SoundPolygon (**B**). In each trial, the subject simultaneously hears a sound (antecedent) and sees two faces (consequents) on the left and right side of the screen. Then the subject guesses which consequent belongs to the given antecedent sound by pressing the “left” or “right” button. The choice is indicated with the green circle. Immediate visual feedback is given. A green checkmark with the word Helyes! (Correct!) indicates a correct guess and a red X with the word Helytelen! (Incorrect!) indicates an incorrect guess.

SoundFace is based on RAET as described in Myers et al.^7,34^. The structure of RAET was kept, but it was translated into the Hungarian language and transformed into an audiovisual paradigm in Assembly for Windows. These modifications were performed with the written permission of Catherine E. Myers. In SoundFace, the subject is first asked to learn associations through trial and error. There are four sounds as antecedents and four possible faces as consequents. The antecedent stimuli are different and clearly distinguishable sounds: a cat (A1), a guitar note (A2), the sound of a vehicle (B1) and a woman’s voice (B2). The consequents are different cartoon faces: an adult male (X1), a girl (X2), an adult woman (Y1), and a boy (Y2). The auditory and visual stimuli were semantically incongruent (except for the case when a woman’s voice is matched with a woman’s face, but this is not always the case). In each trial, the subject simultaneously hears a sound and sees two faces on the left and right sides of the screen. The subject is instructed to guess which face belongs to the given sound and indicate his or her guess by pressing either the „left” or the „right” button. The duration of the auditory stimulus was consistently 1.5 s. The visual stimuli lasted until the participant made the decision with the pressing of the “left” or “right” button. In this respect, SoundFace and SoundPolygon are identical (Fig. 3). The pairs are randomly generated by the software for each subject.

The paradigm is divided into two main phases: the acquisition and test phases. In the acquisition phase, visual feedback was given about the correctness of the choice. In the initial part of the acquisition phase, the subject learns through trial-and-error that if sounds A1 or A2 are presented, the correct response is to choose face X1 over Y1. Similarly, if sounds B1 or B2 are presented, the correct response is to choose face Y1 over X1. This way, it is learned that in terms of their consequents, A1 = A2 and B1 = B2. Once this has been established, new stimulus pairs are added. This time, the subject learns that if sound A1 is presented, the correct response is to choose X2 over Y2, and if sound B1 is presented, the correct response is to choose Y2 over X2. This way, antecedents A1 and B1 gain additional consequents. At this point, the subject knows that A1: X1, X2 and B1:Y1, Y2. Six items are presented in the acquisition phase from the eight possible stimulus pairs. A2:X2 and B2:Y2 are not presented, but it is implied by the connection A1 = A2 and B1 = B2. After each newly introduced stimulus pair, the participant must give a certain number of subsequent correct answers (4, 6, 8, 10, 12 after each new association, respectively) to accomplish the acquisition phase. Because of this, the number of trials in this phase is not constant, and it depends on how efficiently the given individual learns.

**Figure 4:**
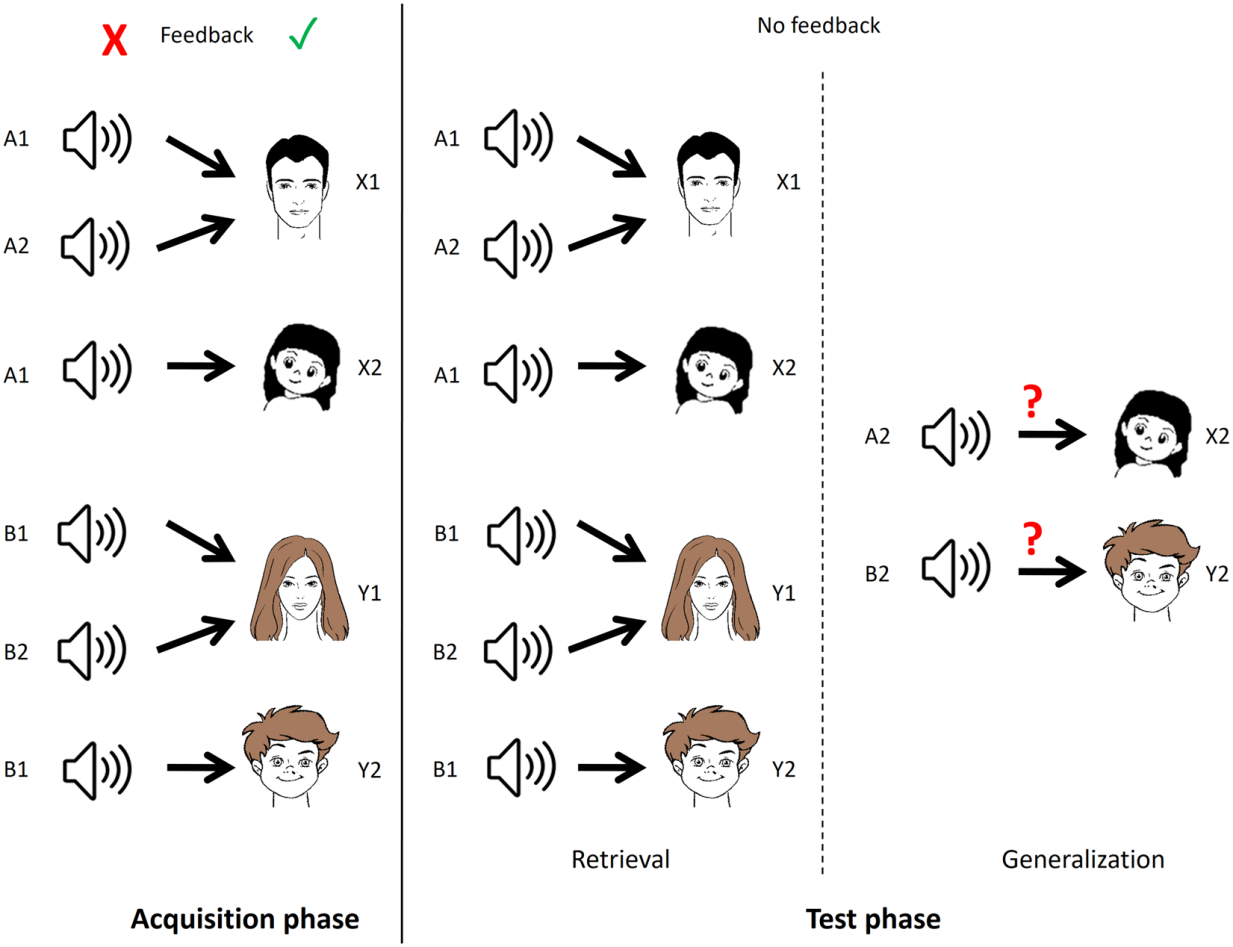
Overview of the structure of the SoundFace test. The antecedent stimuli are sounds of a cat (A1), a guitar note (A2), the sound of a vehicle (B1), and a woman’s voice (B2). The consequents are cartoon faces of a man (X1), a girl (X2), a woman (Y1), and a boy (Y2).

Once having completed the acquisition phase, the participant continues with the test phase, where feedback is no longer given about the correctness of the responses. In this phase, the retrieval and generalization are tested. Retrieval refers to the recall of the already known (learned) stimulus pairs, while generalization refers to making the A2:X2 and B2:Y2 stimulus pairs not presented in the acquisition phase but implied by the connection A1 = A2 and B1 = B2. If the subject has successfully acquired the said associations, he or she will choose X2 when A2 is presented and Y2 when B2 is presented, even if he or she has not seen these pairs before. The subject is not informed that new stimulus pairs are to be expected in the test phase. The number of trials in the test phase is constant. There are altogether 48 trials, of which 36 are retrieval trials, and 12 are generalization trials. These are mixed in random order. The overview of the paradigm is given in Fig. 4. See also Table 1 for clarification.

**Table 1:** A summary of the audiovisual associative learning paradigms.

Acquisition			Test	
Shaping	Equivalence training	New consequents	Retrieval	Generalization
A1—> X1	A1—> X1	A1—> X1	A1—> X1	
	A2—> X1	A2—> X1	A2—> X1	
		A1—> X2	A1—> X2	
				A2—> X2
B1—> Y1	B1—> Y1	B1—> Y1	B1—> Y1	
	B2—> Y1	B2—> Y1	B2—> Y1	
		B1—> Y2	B1—> Y2	
				B2—> Y2

A,B: antecedents (the same sounds in both tests), X,Y: consequents (faces in SoundFace and simple geometric forms in SoundPolygon tests).

**Figure 5:**
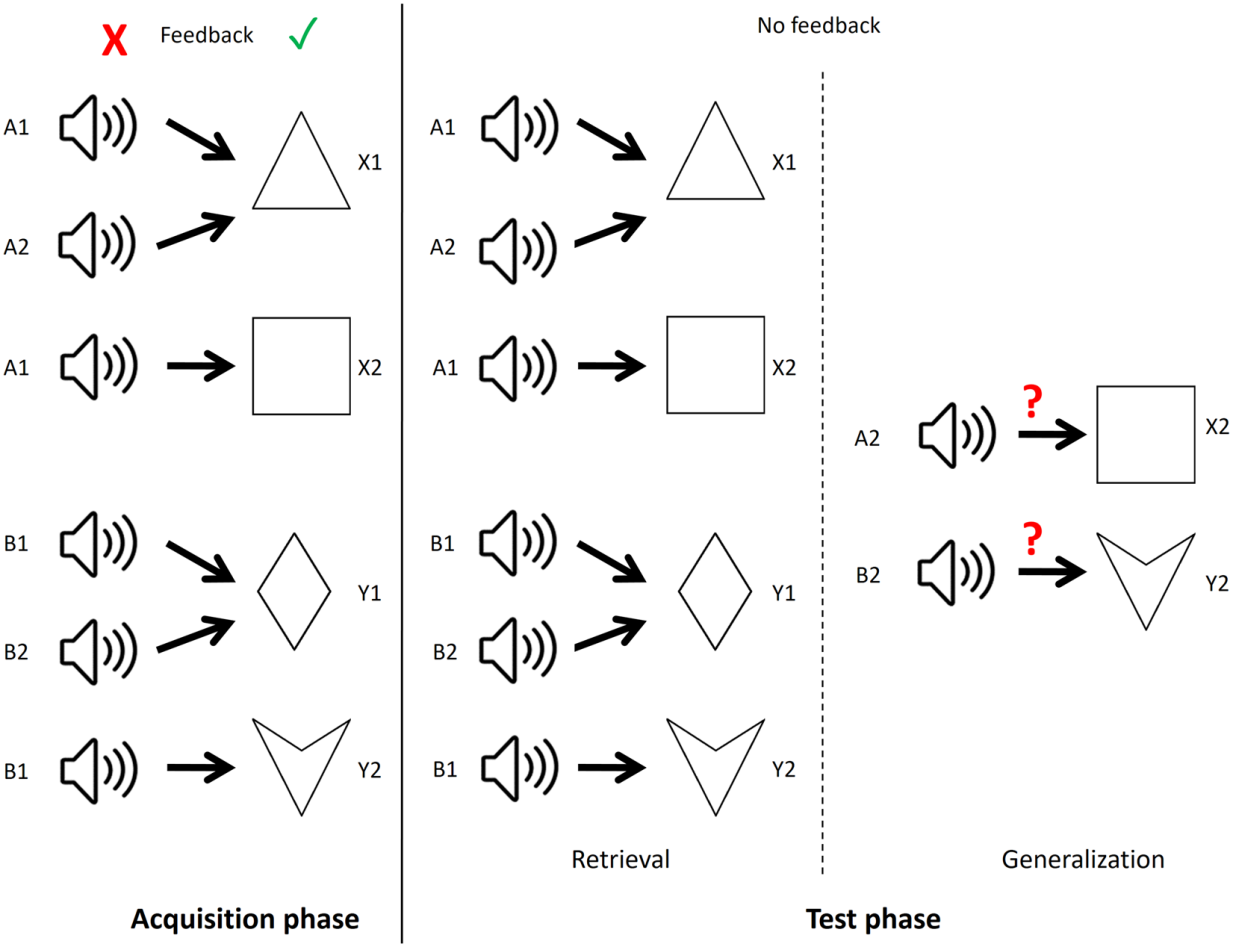
Overview of the structure of the SoundPolygon test. The consequents are simple geometric shapes: a triangle (X1), a square (X2), a rhombus (Y1), and a concave deltoid (Y2).

SoundPolygon has the same structure as SoundFace, but with simplified visual stimuli. Instead of cartoon faces, simple geometric shapes are used as consequents: a triangle (X1), a square (X2), a rhombus (Y1), and a concave deltoid (Y2). The auditory stimuli are the same as in SoundFace. Figure 5 summarizes SoundPolygon.

### Data analysis

The performance of the participants was characterized by four main parameters: the number of trials necessary for the completion of the acquisition phase (NAT), association learning error ratio (the ratio of incorrect choices during the acquisition trials, ALER), retrieval error ratio (RER), and generalization error ratio (GER). NAT and ALER are performance parameters of the acquisition phase. RER and GER are performance parameters of the test phase. Error ratios were calculated by dividing the number of incorrect responses by the total number of trials. Reaction times were recorded for each trial, and they were analyzed for the acquisition, retrieval, and generalization trials separately. Reaction time was defined as the time elapsed between the appearance of the stimuli and the subject’s response. Only RTs of the correct choices were included, and values over 3SD were excluded.

Statistical analysis was performed in Statistica 13.4.0.14 (TIBCO Software Inc., USA). NAT, ALER, RER, and GER were compared between the two paradigms. As the data were non-normally distributed (Shapiro–Wilk *p* < 0.05), the Wilcoxon matched-pairs test was used for the hypothesis tests.

## Supplementary Information

Supplementary Information.

## Data Availability

All data generated or analysed during this study are included in this published article [and its supplementary information files].
